# Comparison of a Lateral Flow Assay and a Latex Agglutination Test for the Diagnosis of *Cryptococcus Neoformans* Infection

**DOI:** 10.1007/s00284-021-02664-w

**Published:** 2021-09-28

**Authors:** Thilo Schub, Johannes Forster, Sebastian Suerbaum, Johannes Wagener, Karl Dichtl

**Affiliations:** 1 Max von Pettenkofer-Institut, Medizinische Fakultät, LMU München, Pettenkoferstr. 9a, 80336 Munich, Germany; 2 Institut für Hygiene und Mikrobiologie, Julius-Maximilians-Universität Würzburg, Würzburg, Germany; 3 Nationales Referenzzentrum für Invasive Pilzinfektionen (NRZMyk), Jena, Germany; 4 Department of Clinical Microbiology, Trinity College Dublin, St James’s Hospital Campus, Dublin, Ireland

## Abstract

Infections by the basidiomycete yeast *Cryptococcus neoformans* are life-threatening diseases claiming more than 600,000 lives every year. The most common manifestation is cryptococcal meningitis in AIDS patients. Diagnosis primarily relies on antigen testing from serum and cerebrospinal fluid (CSF). Current guidelines recommend rapid antigen testing with a focus on point-of-care assays. Over the recent years, a range of new lateral flow assays (LFAs) was launched. There is still a lack of data evaluating the CE-certified Biosynex RDT CryptoPS LFA. We compared the performance of this LFA with a latex agglutination assay (LAA; Latex-Cryptococcus Antigen Detection System, IMMY) from blood and CSF samples. Blood and/or CSF samples of 27 patients with proven cryptococcal infections caused by different species and blood–CSF pairs of 20 controls were tested applying LFA and LAA. Upon combined analysis of blood and CSF, both assays were able to identify all *C.* *neoformans* infections. Based on CSF analysis only, the LFA and the LAA had sensitivities of 100% and 93%. Neither test gave false-positive results nor was reactive in two cases of *C.* non-*neoformans*/non-*gattii* species infections. Both assays have high sensitivities and specificities for the diagnosis of *C.* *neoformans* infection. Contrarily to the IMMY LAA, the RDT CryptoPS LFA is suitable as a point-of-care test but is limited in the quantification of antigen reactivity.

**Supplementary Information:**

The online version contains supplementary material available at 10.1007/s00284-021-02664-w.

## Introduction

Invasive fungal infections claim more lives every year than any other infectious disease except tuberculosis [1, 2]. Almost half of the 1.5 million deaths caused by invasive mycoses are caused by a single fungal genus: the basidiomycete yeast *Cryptococcus*. In the past, this genus was subject of extensive taxonomic reclassification, reducing this previously highly polyphyletic group from more than 100 to now ten species [3, 4]. Even though at least seven species are commonly recognised as pathogens, the major burden of disease can be attributed to *C.* *neoformans* [1, 2, 5, 6].

Immunosuppressed and immunocompromised patients are at particular risk of cryptococcal infections [2, 7]. Individuals suffering from advanced HIV infection are mostly endangered: cryptococcal meningitis (CM) is one of the most common AIDS-defining diseases and accounts for up to 15% of all AIDS-related deaths [8].

In immunocompetent individuals, the infection with the aerogenously transmitted pathogen is usually overcome quickly [9]. In contrast, impaired immunity can lead to the classic clinical picture of CM, with mortality rates ranging from 20 to 70% despite therapy [10]. The outcome largely depends on early onset of targeted therapy [11]. Antifungal treatment of systemic *Cryptococcus* infections is intensive and lengthy: an induction phase of up to several weeks based on the combination of amphotericin B and flucytosine is followed by the consolidation phase over at least the next eight weeks, in which high-dose fluconazole is administered. In reduced doses, this therapy is continued in the maintenance phase/secondary prophylaxis until immune reconstitution or, if necessary, for life [12, 13].

The prerequisite for early therapy initiation is rapid diagnosis. Due to their low sensitivity and specificity, clinical, laboratory chemical and imaging findings cannot provide robust evidence of cryptococcal disease [14]. Microbiological techniques represent the diagnostic gold standard with the ability to proof the infection [13–15]. In the context of CM, the entire spectrum of microbiological diagnostics is covered: microscopy of cerebrospinal fluid (CSF) using India ink, culture, serology, and, with limitations, molecular techniques are in routine use. Due to convenient handling, short turn-around time, and excellent test performance particularly in AIDS patients, the serological detection of cryptococcal antigen is the diagnostic key technique [13–15].

The antigen is part of the cryptococcal capsule, which is a central virulence factor of the fungus. The detected polysaccharide structure is glucuronoxylomannan, which is also released during growth [16]. Differences in the xylose substitutions on its mannose backbone characterise the different serotypes, e.g. capsular antigens A and D for *C.* *neoformans* var. *grubii* and for var. *neoformans* [5].

In this study, we evaluated the performance of the RDT CryptoPS lateral flow assay (LFA) for the detection of cryptococcal capsule antigen in a European cohort. Furthermore, we compared our findings with the results of an established latex agglutination assay (LAA).

## Patients, Materials, and Methods

### Patients and Samples

This retrospective study was performed at the Max von Pettenkofer-Institute for Hygiene and Medical Microbiology that hosts the central microbiology laboratory for the University Hospital of Ludwig-Maximilians-Universität (LMU) in Munich, Germany, and at the Institute for Hygiene and Microbiology that hosts the central microbiology laboratory for the university hospitals of Würzburg (Universitätsklinikum Würzburg). Serum samples of nine additional cases were provided by the German consultant laboratory for cryptococcosis at the Robert Koch-Institute (Berlin, Germany) in the setting of periodical external quality assessments. A total of 39 CSF and 51 blood samples (50 sera and one plasma) of 45 individuals was included in this study (storage at − 20 °C for up to eight years). Twenty-seven patients were diagnosed with invasive cryptococcal infection according to the revised consensus definitions of the EORTC/MSG study group [17].

The control group consisted of twenty consecutive outpatients who underwent CSF puncture in order to exclude neuroborreliosis as cause of neurologic or psychiatric disorders.

### Materials and Methods

All blood and CSF samples were analysed using the Latex-Cryptococcus Antigen Detection System (IMMY, Norman, OK, USA) and the RDT CryptoPS LFA (Biosynex, Illkirch-Graffenstaden, France/Bio-Rad, Hercules, CA, USA). Both tests were performed according to the manufacturers’ instructions. Samples were independently analysed by two trained examiners, and no incongruences occurred. The results of the LAA were read out in titre levels. The LFA allows the differentiation between negative, positive (≥ 2.5 ng/ml capsular antigen), and strong positive results (≥ 25 ng/ml capsular antigen).

Identification of fungal isolates was performed using the MALDI-Biotyper system (Bruker Daltonik, Bremen, Germany). For isolates other than *C.* *neoformans*, identification was performed via sequence analysis of a PCR amplicon (primers: ITS4 and ITS5) of a ribosomal DNA locus (rDNA).

Clinical information and reference standards results were not available to the performers and readers of the assay.

Statistical analysis of measurement distribution using the Mann–Whitney test was performed using Graphpad Prism 5 (GraphPad Software, La Jolla, CA, USA) with an α-level of 0.05 assumed to be significant. The GraphPad QuickCalcs online tool was applied to quantify agreement of measurement results. The calculated kappa index was assessed according to Landis and Koch [18].

## Results

For the analysis of sensitivity, we analysed the specimens (blood and CSF, respectively) which were obtained closest to the day of proven diagnosis (hereafter named day 0). Day 0 was defined as date of sampling of the specimen which allowed the diagnosis of a proven cryptococcal infection according to the EORTC/MSG consensus guideline [17]. In cases, in which this information was available, the mean and median distance between day 0 and the corresponding samples were seven and two days for CSF specimens and zero and two days for blood samples, respectively.

If available, the demographic characteristics, underlying diseases, and the site of infection are summarised in Table 1. HIV infection was the most common precondition and meningitis the most common manifestation of cryptococcal disease (72% and 56%, respectively). *C.* *neoformans* species were cultivated in thirteen cases. There was no infection with species of the *C.* *gattii* complex.

**Table 1:** Demographic characteristics and clinical data

	*n*	%
*C. neoformans* infection	25	
Sex		
Male	12	48
Data not available	9	36
Mean age (if available to laboratory)	45	
Underlying disease		
HIV infection	19	76
Immunosuppressive therapy	3	12
Congenital immunodeficiency	1	4
Risk factors not identified	1	4
Information not available	1	4
Focus of infection		
Meningitis	14	56
Blood stream infection	2	8
Data not available	9	36
Evidence for proven IFI		
Culture positivity	13	52
CSF antigen positivity	12	48
Non-*Cryptococcus* basidiomycete yeast infection	2	5
Male sex	2	100
Mean age (in years)	41	
Characteristics of infection		
Meningitis by *N. albida*	1	50
Wound infection by *F. magnum*	1	50
Evidence for proven IFI		
Culture positivity	2	100
No evidence of cryptococcosis	20	
Male sex	11	55
Mean age (in years)	58	
Indication for lumbar puncture		
Psychiatric assessment	18	90
Neurological assessment	2	10

Non-*Cryptococcus* basidiomycete yeast infections included in this study were caused by *Naganishia albida* (formerly classified as *C. albidus*) and *Filobasidium magnum* (formerly classified as *C.* *magnus*)

**Fig. 1:**
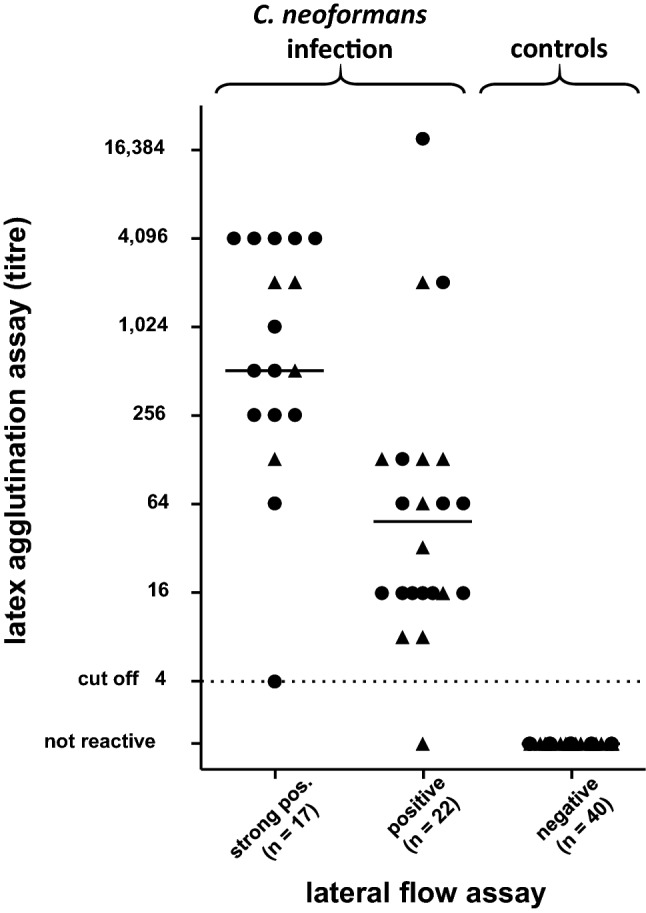
Cryptococcal antigen testing. One serum (circle) and, if available, one CSF (triangle) sample of each case were tested for cryptococcal antigen using the IMMY latex agglutination or the CryptoPS lateral flow assay, respectively. Results (titres) of the agglutination assay were plotted and grouped according to LFA results. The median of each group is indicated. The dotted line represents the cut-off of the latex agglutination assay (titre of 1:4)

All blood samples of *C.* *neoformans*-infected individuals were tested positive by both assays (Table 2, Fig. 1). Regarding the CSF specimens sampled closest to day 0, the latex agglutination assay yielded results above the cut-off in thirteen of fourteen cases (sensitivity of 93%), whilst the LFA was positive in all fourteen cases (sensitivity of 100%). Notably, the patient with the LAA false-negative CSF sample was also only LAA seropositive at the level of the cut-off (titre of 1:4), whilst the LFA yielded a strong positive result. Twenty-nine percent of CSF (4 / 14) and 52% of blood samples (13 / 25) were tested “strong positive”. The group of “strong positive” results was characterised by significantly higher LAA titres (*P* < 0.002).

**Table 2:** Sensitivities and specificities

	latex aggl	LFA
Sensitivity		
Serum	100	100
CSF	93	100
Serum + CSF^a^	100	100
Specificity		
*N. albida* / *F. magnum* infection		
Serum	100	100
CSF	100	100
Serum + CSF	100	100
No evidence of cryptococcosis		
Serum	100	100
CSF	100	100
Serum + CSF	100	100

*Latex aggl.* latex agglutination assay, *LFA* lateral flow assay, *CSF* cerebrospinal fluid

^a^The test was considered to be positive if at least one specimen was tested positive. The combination of serum and CSF was not available in all cases

**Fig. 2:**
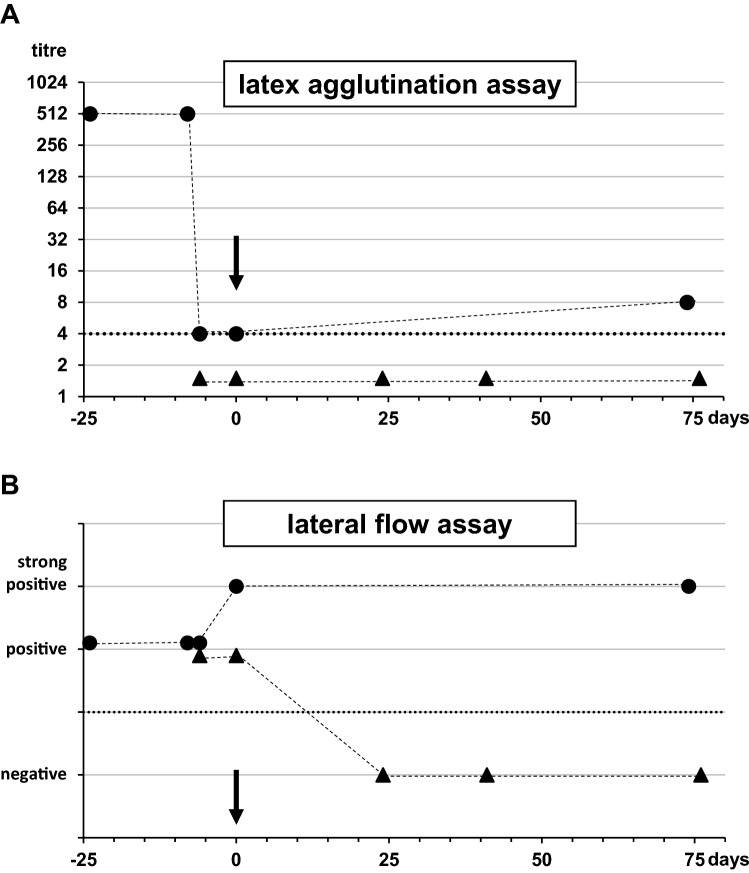
Comparison of the latex agglutination test and the lateral flow assay analysing consecutive samples of a case of cryptococcal meningitis. Serum (circles) and CSF (triangles) samples of the only case with discordant CSF measurement results were analysed with the IMMY latex agglutination assay (**A**) and the CryptoPS lateral flow assay (**B**), respectively. The X-axis depicts the time course with 0 marking the day of CSF sampling resulting in the first cultural and PCR proof of cryptococcal meningitis (arrows). Dotted lines indicate the cut-off (titre of 1:4 and presence/absence of test line) of the assays. Dashed lines illustrate the course of measurement results. No serum or CSF specimen sampled between the analysed specimens was available

The overall agreement between the two assays was almost perfect (kappa = 0.96). The only case with a conflicting result in CSF (positive LFA vs. negative LAA) was subjected to a more detailed analysis (Fig. 2). At the time point of infection, the 41-year-old male received immunosuppressive therapy due to kidney transplantation several years ago. A prozone effect as cause of false negativity was excluded in the respective CSF by repeated measurements with diluted specimen. Next, we identified additional samples from the patient that had been collected in the days and weeks before and after day 0. Interestingly, serum samples from the weeks before day 0 were highly positive for LAA, but then decreased significantly to values to the level of the cut-off. Contrarily, the LFA delivered consistently positive to strong positive results in the available sera. All available CSF samples were antigen negative in the LAA. A seroconversion towards negative CSF results in the further course of the disease was also observed in the (initially positive) LFA.

Additionally, two cases of invasive yeast infections caused by non-cryptococcal basidiomycetes were included: *Naganishia albida* (formerly classified as *C.* *albidus*) was isolated from CSF of a 75-year-old meningitis patient and *Filobasidium magnum* (formerly classified as *C.* *magnus*) was isolated from a wound supposed to communicate with the lumbar canal in a 6-year-old patient with a suspicion of meningitis [3]. Both assays yielded negative results analysing the respective samples. The specificity of both tests was 100% in a control group consisting of corresponding CSF and serum samples of twenty consecutive cases without suspicion of cryptococcosis.

## Discussion

All current guidelines on clinical management of crypotcoccosis recommend antigen testing from CSF to be the primary diagnostic approach in the setting of CM [13–15]. Notably, the WHO guideline for the diagnosis, prevention, and management of *C.* *neoformans* infection 2018 particularly recommends the use of LFAs omitting other serologic approaches [13]. However, this statement is explicitly justified by the epidemiological background of the infection: Due to the high incidence of cryptococcosis especially in developing countries, the WHO guideline considers the diagnostic challenges in a setting with minimal laboratory infrastructure. Addressing this issue, the WHO focussed on tests (and thus especially on LFAs) which can be performed in a resource-limited setting, e.g. without refrigerated storage, laboratory equipment, or skilled technicians.

Alternative methods for the diagnosis of cryptococcosis are characterised by major drawbacks: India ink staining of CSF is compromised by lower sensitivity of only 90% compared to antigen testing [13]. Cultivation of *Cryptococcus* spp*.* is time-consuming and hence cannot provide timely results in an acute clinical setting. *Cryptococcus-*specific PCRs represent promising diagnostic tools, but to date, there is still a lack of robust studies that evaluate the diagnostic performance [19, 20]. Notably, Liesman and colleagues reported an overall positive agreement of only 52% between a multiplex PCR panel and cryptococcal antigen testing [21]. However, the authors of this study speculate that the surprisingly low PCR sensitivity might be attributable to the ability of the antigen to persist in the CSF for a long time even after the infection has been overcome [21, 22].

A number of different antigen tests are currently available (supplementary Table 1). Established antigen tests are characterised by high sensitivity (93%–100%) and specificity (94%–100%) [13, 23]. However, data analysing the performance of the CryptoPS LFA are still limited: three recent studies from Sub-Saharan Africa are available, which all compared the novel CryptoPS LFA to the IMMY CrAg LFA in the setting of HIV-positive patients. In a prospective study relying on a cohort of Botswanan HIV patients with < 200 CD4 cells/µl, Tenforde and colleagues determined the CryptoPS sensitivity to be only 61% (specificity: 97%) [24]. All supposed false-negative samples were characterised by low-level positivity of the IMMY CrAg LFA [24]. However, this LFA was also applied as reference standard, and data on a comparison of the LFAs to the results of culture or molecular testing are not available. Skipper and colleagues retrospectively compared the performance of the CryptoPS LFA and of a prototype semiquantitative LFA to the IMMY CrAg LFA, which was again used as reference standard [25]. In the cohort of 99 sera of Ugandan HIV patients, the CryptoPS LFA was found to have a sensitivity and specificity of 88% and 95%, respectively. However, objective of this study was to identify antigenemia in HIV-infected individuals but not invasive fungal disease. Hence, sera of the positive cohort (*n* = 57) were included regardless of clinical data, and none of the patients had diagnosed CM at the time of sample collection. Temfack and colleagues compared the assay with the IMMY CrAg LFA in a prospective serum-based screening of 186 HIV patients in Cameroon identifying five episodes of CM, which were detected by both tests [26]. Considerably, of all three aforementioned studies, these five infections were the only cases, in which *Cryptococcus* was directly detected. Hence, more data on the performance of the CryptoPS LFA are urgently needed. Therefore, the present study, although also based on a small number of positive cases (13 culture-proven and 12 antigen-proven infections), contributes to our understanding of current antigen tests. Interestingly, the present results indicate higher sensitivity and specificity than observed in the studies by Tenforde and Skipper, which are based on significantly larger study populations. Considerably, the respective cohorts are exclusively characterised for absence or presence of cryptococcal infection by the results of blood tested via the IMMY CrAg LFA. Contrarily, our cohort is additionally based on CSF antigen positivity and cultural findings. One might speculate that this inclusion bias could contribute to the observed differences with our results. A very recent in vitro study analysing homogenised fungal cultures demonstrated that the CryptoPS LFA does not consistently detect pathogenic *Cryptococcus* species that are serotype B or C [27]. The major reason for this deficiency is that only four *Cryptococcus* serotypes were used to setup this assay, whilst there are seven human pathogenic *Cryptococcus* species, which should be considered in the diagnosis of cryptococcal disease [26]. This highlights the impact of the local epidemiology on the performance of the assay. Differences in the prevalence of non-A/non-D *Cryptococcus*, which is less frequently identified from clinical samples in Europe than in Africa [6], might be another explanation for the observed differences in LFA sensitivity between the current study and the studies performed in Sub-Saharan Africa [24–26]. Whilst those suggested a lower sensitivity of the CryptoPS LFA compared to the IMMY CrAg LFA, we even identified one CryptoPS-positive CSF sample tested negative by the IMMY LAA. However, this should not be overrated as antigen detection from serum also allowed for the diagnosis of cryptococcosis in this case. Additionally, alternative techniques including India ink microscopy, different PCR systems (Biofire ME [Biomérieux, Marcy-l’Étoile, France] and an in-house PCR), and even culture (which became positive in a follow-up specimen) also remained negative in this CSF sample.

Whilst the performance of the LFA and the LAA was demonstrated to be comparable, there are significant differences concerning processing of the specimens. The LFA is suited for point-of-care use and can be easily used for a bedside application: CSF and whole blood can directly be transferred to the test device (20 µl). Results can be evaluated after ten minutes. In contrast, the latex agglutination assay requires more elaborated preparation of samples: both CSF and blood must be centrifuged and further pre-treated, including boiling of CSF or pronase treatment at 56 °C of serum. Consequently, laboratory equipment like centrifuge, heating devices, and pipettes are necessary which impedes usage in the clinical setting. However, whilst the LFA only allows to discriminate between the results “positive” and “strong positive”, the LAA provides quantitative results (titres) which may be dispensable in the initial diagnosis but are helpful predictors for survival and CNS involvement, thereby guiding further diagnostic and therapeutic management [28, 29].

## Conclusion

This results in a different field of application for the two tests: In the initial screening (applicable in the clinic as well as in the laboratory), the LFA provides a qualitative result quickly and easily. A positive result should be quantified using the LAA providing an initial value to which the follow-up samples are compared. Testing all subsequent specimens with quantitative assays, i.e. LAA kits, allows treatment monitoring. In this setting, qualitative tests are not beneficial any more. A combination of both approaches has the potential to simplify and even accelerate workflows in the laboratory and/or clinic without compromising the quality of diagnostics.

## Supplementary Information

Below is the link to the electronic supplementary material.

Supplementary file1 (DOCX 17 kb)

## Data Availability

Not applicable.
