# Supplementary material for: Comparison of a Lateral Flow Assay and a Latex Agglutination Test for the Diagnosis of Cryptococcus Neoformans Infection
Source: Curr Microbiol. 2021 Sep 28;78(11):3989–95. doi: 10.1007/s00284-021-02664-w (PMC8486725; doi:10.1007/s00284-021-02664-w)
Supplement: Supplementary file 1 — Supplementary file1 (DOCX 17 kb) [file 284_2021_2664_MOESM1_ESM.docx]

**Supplementary table 1. Commercially available *Cryptococcus* antigen tests.**

LAA latex agglutination assay; ELISA enzyme linked immunosorbent assay; LFA lateral flow assay;

|  | **assay** | **manufacturer** |
| --- | --- | --- |
| **LAAs** | |  |
|  | CALAS | Meridian Biosciences |
|  | Crypto-LA | International Biological Labs |
|  | IMMY CrAg | IMMY |
|  | Remel / Murex CrAg test | Remel |
|  | Pastorex | Bio-Rad Laboratories |
|  | Serodirect | Eiken |
| **ELISAs** | |  |
|  | ALPHA CrAg ELISA | IMMY |
|  | *C. neoformans* Ag ELISA | Dynamiker Biotechnology |
|  | Premier CrAg ELISA | Meridian Biosciences |
| **LFAs** | |  |
|  | Cryptococcal Capsular Polysaccharide K-Set | FungiXpert |
|  | Dynamiker CrAg LFA | Dynamiker Biotechnology |
|  | IMMY CrAg LFA | IMMY |
|  | RDT CryptoPS | Biosynex |
|  | StrongStep CrAg Rapid Test | LimingBio |
